# Decoupling of Elderly Healthcare Demand and Expenditure in China

**DOI:** 10.3390/healthcare9101346

**Published:** 2021-10-10

**Authors:** Shangguang Yang, Danyang Wang, Wenhui Li, Chunlan Wang, Xi Yang, Kevin Lo

**Affiliations:** 1 Economic Development Institute, East China University of Science and Technology, Shanghai 200237, China; sgyang@ecust.edu.cn; 2 School of Business, East China University of Science and Technology, Shanghai 200237, China; livialwh@163.com; 3 Chinese Modern City Research Center, School of Social Development, East China Normal University, Shanghai 200062, China; clwang@soci.ecnu.edu.cn; 4 David C. Lam Institute for East-West Studies, Hong Kong Baptist University, Hong Kong 999077, China; xiyang@hkbu.edu.hk; 5 Department of Geography, Hong Kong Baptist University, Hong Kong 999077, China

**Keywords:** population aging, healthcare demand, healthcare expenditure, China

## Abstract

This study examined the changing trajectory and factors that influenced the health and medical expenditure of the Chinese elderly population over the past two decades. Based on the Chinese Longitudinal Healthy Longevity Survey (CLHLS) from 1998 to 2018, inferential and multiple linear regression models were constructed. The key finding is that China has experienced a decoupling of healthcare demand (HCD) and healthcare expenditure (HCE) since around 2014, when HCE began to decline despite the fact that HCD continued to rise. This is a promising sign, suggesting that the government’s health insurance policy is working. Furthermore, participating in health insurance schemes can significantly reduce the elderly’s HCD and HCE, demonstrating that health insurance can effectively affect the elderly’s decision to seek medical treatment and improve their health condition. We also found that age, region, basic old-age insurance, and care by the government and institutions were significant factors that influenced the healthcare demand and expenditure of the elderly population.

## 1. Introduction

An aging population is one of the most important medical and healthcare challenges worldwide, and China is no exception. As the world’s most populous country, China’s aging process surpasses the world average. The number of people over 60 years of age reached 254 million in 2019, accounting for 18.2% of the Chinese population [1]. This means that China has the largest elderly population worldwide [2]. The healthcare demand (HCD) and healthcare expenditure (HCE) of the elderly are evolving along the process of reforming China’s healthcare system [3]. It is predicted that the percentage of GDP spent on pensions, healthcare, welfare, and facilities will increase from 7.33% in 2015 to 26.24% in 2050 [4]. However, the literature on the HCD and HCE for the Chinese elderly population is limited [5,6]. In particular, existing studies typically use cross-sectional data and are therefore unable to analyze changes in the elderly population’s HCD and HCE over time. This is an important research gap for countries facing dramatic socioeconomic and policy changes, which may profoundly shape the elderly population’s HCD and HCE.

Responding to the challenge of healthy aging, this study aims to generate knowledge regarding the healthcare demand and out-of-pocket healthcare expenditure of the elderly population. We employed Grossman’s health demand model to classify the factors of HCD and HCE. Grossman’s health demand model is an influential analytical framework for investigating HCD and HCE [7]. This model introduced the concept of health as “a durable capital stock that yields an output of healthy time”, and the price of health depended on several variables, such as healthcare services, income, education, age, gender, and so on. In addition, an increase in the price may simultaneously reduce the quantity of HCD and increase the demand for healthcare inputs [8]. The data for this empirical study were derived from the Chinese Longitudinal Healthy Longevity Survey [9]. CLHLS collected longitudinal data coordinated by the Center for Healthy Aging and Development Studies of the National School of Development at Peking University. The baseline and follow-up surveys were conducted in eight waves: 1998, 2000, 2002, 2005, 2008–2009, 2011–2012, 2014, and 2017–2018. A total of 113,000 face-to-face home-based interviews were conducted between 1998 and 2018. The survey randomly selected about half of the counties and city districts in 23 provinces across the country. As the data covered approximately 85% of the Chinese population, it can be seen as a representative survey among the national elderly [10].

The rest of this paper is organized as follows. A literature review is presented in Section 2. The methods are outlined in Section 3. The results are presented in Section 4 and discussed in Section 5. Lastly, the policy implications of these findings and several limitations are discussed in the conclusion.

## 2. Literature Review

Providing adequate healthcare for the elderly population has become an important challenge in many countries. Meesters et al. [11] assessed the care needs of elderly schizophrenia patients and found that their psychological and social needs appeared to be underserviced, and unmet needs were associated with a decline in quality of life. Stein et al. [12] studied elderly German patients aged 85 years and older with mild cognitive dysfunction and found that the unmet needs of these elderly individuals were associated with mild cognitive impairment and dementia, and the other risk factors were age, education, and marital status. In India, in the context of poverty, isolation, changes in residential care, and weak institutional support, female elderly are the most vulnerable group, especially for elderly widowed women who have lower literacy, limited social exposure, and monetary dependence [13]. In the context of insufficient healthcare, the reciprocal nature of intergenerational support is important, and the elderly make contributions through assistance in household chores, caregiving, and economic activity. These informal systems of resource exchanges within families are important for maintaining the well-being and fulfilling the HCD of the elderly population [14].

In addition, the elderly population often presents multiple diseases, and multimorbidity is the most common clinical problem among the elderly population and may be increased by unhealthy behaviors [15]. Chronic diseases not only impose heavy health and financial burdens on the elderly, families, and healthcare systems, but might also induce psychological diseases over time. Strong, linear associations were found between the number of chronic diseases and psychological symptoms, which indicated that psychological distress among the elderly was more apparent in the presence of more than one disease [16]. For example, the level of depressive symptoms varies across different types of chronic diseases, and depressive symptoms are largely attributable to physical limitations in stroke [17]. Therefore, it is necessary to prevent and recognize early and optimal treatment of chronic diseases in the elderly population by identifying new risk factors and risk indicators of chronic diseases [18]. The development and implementation of models for the prevention and control of chronic diseases in the elderly population have been proposed at the community level under the active aging paradigm [19]. Furthermore, inappropriate medication use has a significant adverse effect on the health status of the elderly population [20].

Furthermore, HCD is affected not only by demography and morbidity-related factors, but also by socio-cultural status. Social relationships exert powerful impacts on the health of the elderly population; for example, they may experience the loss of one or more close relationships, and this usually has an effect on their health and the reorganization of their social ties in later life [21]. Djundeva et al. [22] found that the possibility of having restricted and child-based social networks was greater in Eastern and Southern European countries, whereas people in Western and Northern European countries were likely to have more friend-oriented social networks. In particular, elderly people with diverse social networks have better well-being than those with restricted social networks. In addition, the lack of acceptance of aging, old age, and death in a particular culture makes it difficult for people to realize that caring for the elderly is a natural part of life, which may result in an increased demand for institutional care [23]. 

The impact of accelerated aging on HCE is extremely significant. Functional decline of the elderly population inevitably leads to increased illness and lengths of hospitalization and readmission [24]. Hogan et al. [25] pointed out that medicare outlays in the last year of life accounted for nearly one-quarter of the medicare beneficiaries’ total costs. In addition, HCE rises quickly with age and income, and the risk of requiring expensive medical care is a key driver of saving for many higher-income elderly people [26]. It is believed that the increase in HCE is partly due to the accelerated aging population and the rapid rise in the number of elderly people with disabilities and chronic illness; in particular, HCE will expand rapidly in less developed countries to reach levels currently observed in more developed countries [27]. Along with the aging process and socioeconomic development, the elderly population’s HCD is shaped by influential factors such as accessibility to healthcare resources, living arrangements, health information, and so on [28,29]. Existing studies have demonstrated that both family and community social capital play important roles in influencing HCD and HCE [30]. In addition, the influence of social protection benefits on the intergenerational distribution of resources and private family transfers is recognized in the literature [31].

## 3. Materials and Methods

### 3.1. Data

CLHLS participants included elderly people aged 65 years and above, as well as middle-aged adults aged 35–64 years. The survey provided reliable information on the health status and influencing factors of the elderly from various dimensions, including family structure, living arrangements and proximity to children, activities of daily living (ADL), the capacity of physical performance, self-rated health, self-evaluation of life satisfaction, cognitive functioning, chronic disease prevalence, care needs and costs, social activities, diet, smoking and drinking behaviors, psychological characteristics, economic resources, and care giving and family support among elderly respondents and their relatives.

Our empirical analysis excluded samples that were missing from the system and some that did not meet the variable conditions, such as those who answered “unclear” in the question of whether there was public pension insurance. There were 9051 valid samples in 1998, 10,889 valid samples in 2000, 15,743 valid samples in 2002, 15,447 valid samples in 2005, 16,814 valid samples from 2008 to 2009, 8056 valid samples from 2011 to 2012, 3601 valid samples in 2014, and 12,927 valid samples in 2018. In this study, we first described the HCD and HCE of each period, and then analyzed the evolutionary trends of the corresponding changes over time. We then constructed multiple linear regression models using the IBM SPSS software (version 22.0) (IBM, Armonk, NY, USA).

### 3.2. Model

Based on Grossman’s health demand model and the variables of CLHLS, we chose a multiple linear regression model to empirically analyze the influencing factors of various independent variables on HCD and HCE. This study built the following model:

(1) $$\{\begin{array}{l} y_{i}=\beta_{0}+\beta_{1}x_{i1}+\ldots +\beta_{p}x_{ip}+\varepsilon_{i},i=1,2,\ldots ,n\\ E\left(\varepsilon_{i}\right)=0,\\ D\left(\varepsilon_{i}\right)=\sigma^{2},\\ cov(\varepsilon_{i},\varepsilon_{j})=0,i\neq j,\\ i,j=1,2,\ldots ,n \end{array}$$

where *y_i_* represents the dependent variable. This study has two dependent variables (HCD and HCE), represented by *y_1_* and *y_2_*, respectively; thus, it is divided into two parts for regression. *Xi* represents the independent variable and there are 12 independent variables in total. Among them, *x_1_* to *x_4_* correspond to gender, age, cohabitation status, and residence among the demographic characteristic variables, *x_5_* to *x_10_* correspond to region, education, household income, BOAI, and BMI among the socioeconomic status variables, and *x_11_* to *x_12_* represent living arrangements and number of intergenerational support variables, respectively. As the survey objects of each period are quite different and there was a mixture of cross-sections over time, the model was constructed independently according to the cross-sectional data of each period.

As the CLHLS questionnaire was evolving and updating during this period, several important variables were introduced into the survey since 2005, such as HCE, BOAI, and BMI, and an inflection point that occurred in 2008; thus, we selected the cross-sectional data of 2005 and 2014 for multiple linear regression analysis. The multiple linear regression models with HCD and HCE as the explained variables for each period are as follows:

(2) $$y1=\beta_{0}+\beta_{i}xi+\varepsilon 1$$

(3) $$y2=\beta_{0}+\beta_{i}xi+\varepsilon 2$$

The variance inflation factor (VIF) was used to test the multicollinearity of the multiple regression models. If the VIF exceeds 10, multicollinearity exists, and the corresponding variables should be removed. After testing, no variable had a VIF > 5; thus, there was no multicollinearity between the independent variables of the multiple linear regression models.

### 3.3. Variables

This study assigned the HCD and HCE of the elderly as dependent variables. We selected ADL, a commonly used measure to determine a person’s healthcare need, as the indicator to measure HCD. The measurement of ADL was derived from CLHLS, which included six aspects of the elderly’s daily activities, including (1) bathing and showering, (2) dressing, (3) toilet hygiene, (4) indoor mobility, (5) continence, and (6) eating. For each aspect, respondents were asked to state whether they could perform the activity independently (score = 1), independently but with some help (score = 2), or only with help from others (score = 3). We scored it according to values that ranged from 6 to 18 points, where higher scores indicated more HCD. For elderly HCE, the variable “medical services costs” was introduced in the 2005 survey. The item asked respondents their out-of-pocket medical expenditure (CNY) in the previous year.

According to Grossman’s theory, the influencing factors of HCD include three variables: (1) demographic variables, (2) socioeconomic status variables, and (3) intergenerational support variables. A description of the independent variables and data selection is presented in Table 1. The variable household income was added to the CLHLS survey in this study since 2002, and HCE, BOAI, and BMI were added since 2005. Table S1 shows the mean and standard deviation of each variable for each year’s cross-sectional data.

## 4. Results

### 4.1. Trends and the Influencing Factors of Healthcare Demand and Expenditure

#### 4.1.1. HCD

**Figure 1:**
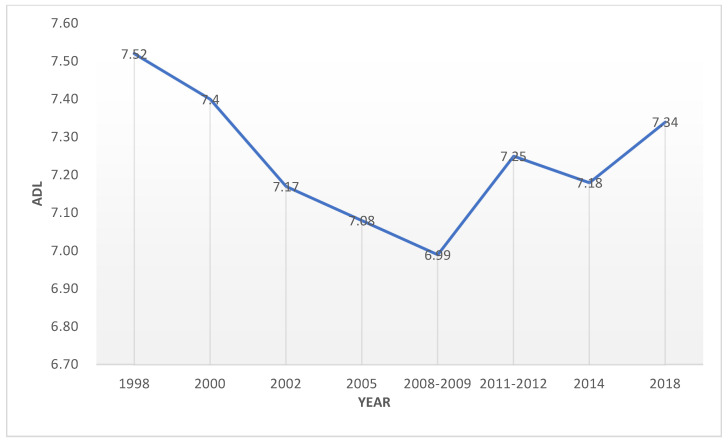
Elderly HCD in China (1998–2018).

Figure 1 shows the average elderly HCD from 1998 to 2018. The HCD fluctuates from 6.99 to 7.52. As we used ADL (activities of daily living) as a proxy to HCD, the values show that in general the respondents have good functional capacity but are dependent on others for one or two activities. Furthermore, temporally, the elderly HCD exhibits a U-shape pattern. The HCD of the elderly population gradually decreased from 1998 to 2008. This might be due to the improvement in the health status of the elderly, which was inseparable from the rapid socioeconomic progress and healthcare service development in China. During the past decade, China’s insurance system has begun to spread, making it more accessible for the elderly to see doctors and receive proper treatment, thus improving their overall health condition. However, there was a turning point in 2008, and the elderly HCD has increased ever since. This might be attributed to the acceleration of population aging, the extension of life expectancy, and the overall decline in the health condition of the elderly.

As for the influencing factors (Table S2), women’s HCD was consistently higher than that of males, and the HCD of the elderly over 80 years old was greater than that of the non-elderly. From the perspective of regional and income divisions, low-income elderly people in rural and underdeveloped areas had low HCD, which was a contrast to the common belief that the population in developed regions should be healthier. This may be correlated with the aging population in developed regions. Furthermore, participating in the BOAI and BMI can significantly reduce HCD, indicating that these insurance policies can effectively protect the health of the elderly. In addition, the HCD of people under the care of the government and institutions was higher than others, which might be because the health conditions of the elderly population are much more vulnerable in general; therefore, they usually require more health and medical services.

#### 4.1.2. HCE

**Figure 2:**
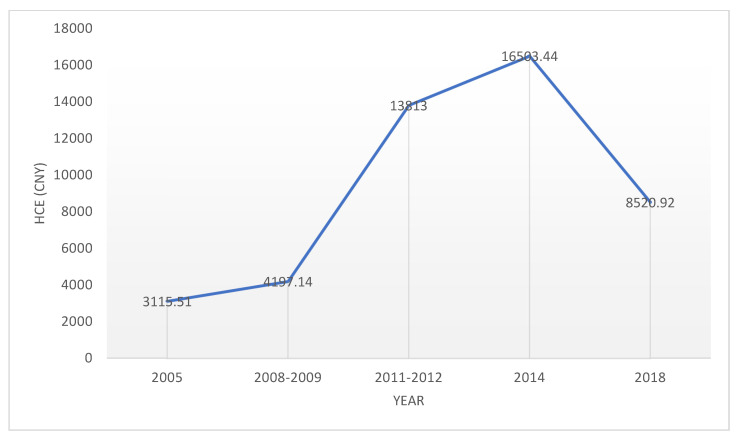
Elderly HCE in China (2005–2018).

Figure 2 plots the average annual HCE (CNY) from 2005 to 2018. The HCE of the elderly exhibits an inverted-U shape, with a clear growth trend from 2005 to 2014, peaking in 2014 at CNY 16503, followed by a sustained decline from 2014 to 2018. This robust decline may be attributed to better government support, including the 50% reduction in the medical insurance threshold and the increase in the reimbursement ratio, which greatly reduced people’s out-of-pocket medical expenditure. Overall, the increasing growth trend of HCE after 2008 is in line with the time trend reflected in the HCD, which proves that the overall health status of the elderly would deteriorate and the medical resources they need would increase in an aging society.

As shown in Table S3, women’s HCE was lower than that of males, despite the fact that their HCE was higher. Furthermore, the eastern region’s HCE was higher than others, which may be related to the fact that the economically developed regions had a higher HCD due to higher levels of population aging, as well as higher costs of healthcare in general. In addition, HCE increased according to income level. In addition, elderly participants in the BOAI and BMI had significantly higher HCE, which indicated that these public insurance policies promoted the elderly to transform their HCD into medical needs at a certain level. The elderly were encouraged to go to the hospital because these insurances could reduce their financial burden. From the perspective of the source of living arrangements, elderly people who were under the care of the government and institutions had higher HCE, which was consistent with the results of HCD.

### 4.2. Multiple Linear Regression on Influencing Factors

#### 4.2.1. HCD

Regarding the factors that influenced elderly HCD (Table S4), among the various demographic factors, gender and HCD had significant negative correlations in the two-period models (*p* < 0.01), which indicated that male elderly had better health conditions than females in general. In addition, there was a significant correlation between age and HCD of the elderly (*p* < 0.01), which indicated that the growing aging population would bring about an increase in HCD.

Regarding socioeconomic factors, the central region had a significantly higher HCD than the western district in the model (*p* < 0.01), and the eastern region had a higher coefficient than the central region in 2014, which suggested that the HCD of the elderly in economically developed areas was higher. In addition, education was positively correlated with HCD, and household income was negatively correlated with HCD but the impact was limited. In 2014, participation in BMI significantly reduced HCD, which indicated that the recent popularization of the public insurance system had improved the health of the elderly.

From the perspective of intergenerational support, there was no significant difference between family caring and the elderly living alone, whereas the HCD of elderly people under the care of the government and institutions was significantly higher than those taking care of themselves.

#### 4.2.2. HCE

As for the factors that influenced the elderly HCE (Table S5), residence had a significantly positive correlation (*p* < 0.01) with HCE, which indicated that the urban HCE had increased by 0.1–0.2% compared with rural areas. From the perspective of socioeconomic status, the central and eastern regions had higher HCE than the western region (*p* < 0.01). The more developed the economy, the larger the correlation coefficient, which indicated that there was a much higher HCE. In addition, the influence of household income was slightly positive. In particular, both BOAI and BMI were significantly correlated with HCE (*p* < 0.01), which indicated that the participation of public insurance significantly altered the HCE of the elderly. In particular, it is worth noting that participation in insurance increased HCE in 2005, whereas it was the opposite in 2014. This is because in the early stage of the introduction of BOAI and BMI, the elderly may have “excessive medical demand”, which leads to higher expenditure. With the improvement in the old-age insurance system and medical insurance system, the government has undertaken more medical expenditure, and the HCE of the elderly will decrease accordingly. Moreover, in terms of intergenerational support, there was no significant difference between family caring and the elderly living alone, whereas the HCE of the elderly under the care of the government and institutions was significantly increased (*p* < 0.01). Along with the growing aging population, this influence was much higher in 2014. Furthermore, the number of children had limited influence on the elderly’s HCE, which suggested that the weakening of intergenerational support had shifted the healthcare burden to government and pension institutions.

## 5. Discussion

This research contributes to the growing literature on the elderly population’s healthcare demands and expenditure by employing a large-scale panel dataset and reveals the temporary trends. The empirical findings of this study can be used to inform public healthcare decision-making and fill gaps in the knowledge base, which leads to a better understanding of how to deal with the aging process and achieve active and healthy aging. There are several implications that will be discussed further in this section.

First, given the growing aging population in China, healthcare demands have shown increasing trends since 2008. These indicators are expected to continue to increase in the future. The rising elderly HCD comes at a time when society is undergoing fundamental changes. Family support has traditionally played an important role in elderly healthcare services in China [32]. However, nowadays, non-traditional families have emerged in large numbers, such as exclusively elderly families, empty-nest families, grandparent families, and so on [33]. The rate of intergenerational co-residence with adult children is declining among the elderly population in China, and the primary living arrangement for them is living close to children [28]. Feng et al. [34] identified a significant influence of neighborhood environments on health-related quality of life in the elderly population. Meanwhile, after the unprecedented “one child” policy, Chinese families are becoming old due to the low birth rate and longer life expectancy [35]. The substantially reduced number of children has shortened the length of family expansion, while increasing the period of empty-nest elderly [32]. The “4-2-1” family structure and the empty nest undermine the traditional Chinese ways of caring for the elderly, such as family support. Thus, social support, such as community-based nursing models and nursing curriculum reforms with a gerontology focus, is becoming more and more important [36]. The elderly care service industry should include community nursing services, elderly care institutions, and other organizations to replace the family to take care of the elderly to a certain extent. To fill in the gap in family-oriented public services and provide effective help to the elderly population, it is necessary for the government to shift the focus from family-based elderly care to social support. The government should accelerate the construction of the elderly care service system to address the increasing pressure on elderly care and HCD. Furthermore, the implication for promoting elderly health is that both primary and secondary prevention initiatives must be taken.

Second, the trends of HCD and HCE are related but can also be different. In China, there was a decoupling of HCE and HCD at around 2014, when HCE began to decline despite the fact that HCD continued to rise. This is a promising sign, suggesting that the government’s health insurance policy is working. To deal with the rapid increase in HCE and guarantee that the elderly receive material assistance, the Chinese government has established a social insurance system consisting of basic old age insurance (BOAI) and basic medical insurance (BMI). These public pension schemes, such as the BOAI, aim to provide basic social security to all elderly residents regardless of whether they were employed, and the BMI is an integrated medical insurance system with nearly universal coverage [37]. Furthermore, the New Rural Pension Scheme was launched in 2009 and covered nearly all counties by 2012. Pension income has significantly reduced intergenerational co-residence and increased independent living in the rural elderly population [38]. Until 2017, Chinese public pension schemes had more than 915 million participants, and the total public pension expenditure was CNY 4032 billion, which is about 5% of China’s GDP [39]. In general, participating in the BOAI and BMI can significantly reduce elderly HCD and HCE, demonstrating that health insurance can effectively affect the elderly’s decision to seek medical treatment and improve their health condition.

Third, this study has some limitations. We were not able to provide wider and more detailed information about the elderly population’s HCD and HCE due to the limitations of the CLHLS structure. In addition, data on factors that indirectly influenced individuals’ HCD and HCE have not been considered, such as the development of the elderly’s health knowledge, individuals’ previous medical experience, doctor–patient relationships, social capital, and so on. HCD and HCE were determined by a complex interplay, which included patient characteristics and providers’ structure, process, and outcome. Thus, future studies should consider these aspects and place more emphasis on a full-scale set of measurements and analyses.

## Figures and Tables

**Table 1:** Assignment of variables.

Type	Variable	Definition
Dependent variables	HCD	ADL score from CLHLS (E1 to E6 in the questionnaire)
	HCE	out-of-pocket healthcare expenditure in the previous year
Demographic variables	gender	female = 0; male = 1
	age	participants’ age in that year
	cohabitation status	living alone = 1; living with family = 2; living in nursing home = 3
	residence	country = 0; city = 1
Socioeconomic variables	region	western = 1; central = 2; eastern = 3
	education	participants’ years of education
	household income	participants’ total household income in the previous year
	income level	low = <30%, medium = 30–70%, high = >70%
	BOAI	participation in BOAI: no = 0; yes = 1
	BMI	participation in BMI: no = 0; yes = 1
Intergenerational support variables	living arrangements	self = 1; family and friends = 2; government and institutions = 3
	children number	participants’ total number of children

## Supplementary Materials

The following are available online at https://www.mdpi.com/article/10.3390/healthcare9101346/s1, Table S1: Descriptive statistics, Table S2: Influencing factors of elderly HCD in China (1998–2018), Table S3: Influencing factors of elderly HCE in China (2005–2018), Table S4: Multiple linear regression on elderly HCD, Table S5: Multiple linear regression on elderly HCE.
