# Supplementary material for: Decoupling of Elderly Healthcare Demand and Expenditure in China
Source: Healthcare (Basel). 2021 Oct 10;9(10):1346. doi: 10.3390/healthcare9101346 (PMC8544591; doi:10.3390/healthcare9101346)
Supplement: Supplementary file 1 [file healthcare-09-01346-s001.zip › healthcare-1383304-supplementary.pdf]

Table S1. Descriptive statistics

Variable	1998 mean	1998 SD	2000 mean	2000 SD	2002 mean	2002 SD	2005 mean	2005 SD	2008-2009 mean	2008-2009 SD	2011-2012 mean	2011-2012 SD	2014 mean	2014 SD	2018 mean	2018 SD
HCD	7.52	2.7	7.4	2.7	7.17	2.4	7.08	2.5	6.99	2.4	7.25	2.7	7.18	2.6	7.34	2.8
HCE	-*	-	-	-	-	-	3115.51	13119.3	4197.14	15618.1	13813	38832.3	16503.44	41585.7	8520.92	27018.6
gender	0.4	0.5	0.41	0.5	0.43	0.5	0.43	0.5	0.43	0.5	0.45	0.5	0.47	0.5	0.43	0.5
age	92.11	7.7	91.28	7.5	86.25	11.7	86.13	11.7	86.83	12	85.87	11.3	84.96	9.7	85.55	11.9
cohabitation status	1.2	0.5	1.25	0.6	1.22	0.5	1.18	0.4	1.18	0.4	1.19	0.4	1.19	0.4	1.88	0.4
residence	0.15	0.4	0.17	0.4	0.16	0.4	0.16	0.4	0.13	0.3	0.11	0.3	0.12	0.3	0.16	0.4
region	1.68	0.7	1.68	0.7	1.66	0.7	1.84	0.8	1.67	0.7	1.66	0.7	1.73	0.7	2.36	0.7
education	2.27	7.7	1.83	3.4	2.02	3.5	2.11	3.5	2.06	3.4	2.33	3.5	2.57	3.6	3.55	6.7
household income	-	-	-	-	7939.58	20786.2	12625.13	25088.6	22518.57	26275.6	30532.93	30309.3	37830.94	32465.8	44709.13	37351.8
BOAI	-	-	-	-	-	-	0.06	0.2	0.07	0.3	0.18	0.4	0.29	0.5	0.3	0.5
BMI	-	-	-	-	-	-	0.1	0.3	0.16	0.4	0.66	0.5	0.65	0.5	0.62	0.5
living arrangements	2.06	0.3	2.06	0.3	2.03	0.3	2.03	0.2	2.01	0.2	2.01	0.2	2	0.2	2.03	0.2
children number	5.94	11.1	4.59	2.7	4.55	2.5	4.5	2.4	4.36	2.2	4.35	2.1	4.37	2	4.22	5.2

* "-" represents unavailable data. Due to the continuous enrichment of CLHLS survey content, there are some unavailable data before 2005

Table S2. Influencing factors of elderly HCD in China (1998-2018)

		**1998**		**2000**		**2002**		**2005**		**2008-2009**		**2011-2012**		**2014**		**2018**	
		**mean**	**%**	**mean**	**%**	**mean**	**%**	**mean**	**%**	**mean**	**%**	**mean**	**%**	**mean**	**%**	**mean**	**%**
Gender	female	7.869	60	7.671	58.7	7.455	57.4	7.343	57.3	7.255	57.3	7.511	54.9	7.405	53.4	7.67	57.1
	male	6.998	40	7.022	41.3	6.779	42.6	6.723	42.7	6.635	42.7	6.93	45.1	6.917	46.6	6.963	42.9
Age	non-elderly	6.376	6.2	6.482	5.5	6.185	32.7	6.189	33.9	6.168	30.3	6.287	35	6.374	39.3	6.208	37.6
	old age	7.596	93.8	7.457	94.5	7.645	67.3	7.535	66.1	7.348	69.7	7.767	65	7.698	60.7	8.064	62.4
Residence	country	7.553	84.8	7.397	83.3	7.141	84.4	7.064	84.5	6.965	87.2	7.219	89.5	7.151	88.5	7.341	83.3
	city	7.338	15.2	7.43	16.7	7.31	15.6	7.159	15.5	7.166	12.8	7.504	10.5	7.381	11.5	7.495	16.7
Region	eastern	7.723	48.3	7.587	47.7	7.288	48.1	7.243	45.3	7.137	46.4	7.363	48.1	7.172	43.9	7.548	50.6
	central	7.33	35.6	7.265	36.4	7.06	37.3	7.135	25.8	6.877	40	7.164	38.2	7.244	39.7	7.125	35.8
	central	7.33	35.6	7.265	36.4	7.06	37.3	7.135	25.8	6.877	40	7.164	38.2	7.244	39.7	7.125	35.8
Income level	low					7.111	31.5	7.022	29.9	6.75	30.1	6.983	27.6	6.963	30.2	7.236	30.8
	medium					7.074	34.4	7.026	36.9	7.031	39.9	7.392	41	7.342	35.4	7.388	37.2
	high					7.314	34.1	7.189	33.1	7.178	30	7.296	31.4	7.196	34.3	7.467	32
BOAI	no							7.062	94.5	6.985	93.1	7.27	82.4	7.221	70.9	7.391	70.3
	yes							7.376	5.5	7.069	6.9	7.151	17.6	7.071	29.1	7.292	29.7
BMI	no							7.089	90.1	6.993	84.5	7.532	33.7	7.4	35.1	7.637	38.3
	yes							6.983	9.9	6.979	15.5	7.106	66.3	7.057	64.9	7.189	61.7
Living arrangements	Self	6.292	1.1	6.312	2	6.27	2	6.1	1.7	6.081	2.1	6.097	1.8	6.085	1.6	6.132	1.7
	family and friends	7.531	92.3	7.367	90.2	7.108	92.6	7.028	94	6.959	95	7.204	95.5	7.159	96.5	7.265	94
	government and institutions	7.612	6.6	8.088	7.8	8.53	5.4	8.584	4.3	8.725	2.9	9.631	2.7	9.059	1.9	10.038	4.3

Table S3. Influencing factors of elderly HCE in China (2005-2018)

		2005		2008-2009		2011-2012		2014		2018	
		**mean**	**%**	**mean**	**%**	**mean**	**%**	**mean**	**%**	**mean**	**%**
Gender	female	2895.994	57.30	4161.032	57.30	13387.733	54.90	16133.612	53.40	8253.46	57.10
	male	3410.462	42.70	4245.634	42.70	14335.528	45.10	16927.267	46.60	9248.637	42.90
Age	non-elderly	3011.427	33.90	3998.284	30.30	11722.107	35.00	14293.72	39.30	8876.945	37.60
	old age	3168.866	66.10	4283.625	69.70	14926.553	65.00	17935.461	60.70	8561.769	62.40
Residence	country	2894.479	84.50	4028.27	87.20	12803.671	89.50	16386.342	88.50	7734.804	83.30
	city	4317.683	15.50	5344.654	12.80	22395.414	10.50	17402.422	11.50	13363.969	16.70
Region	eastern	4335.053	45.30	6384.102	46.40	15569.863	48.10	16496.724	43.90	10724.68	50.60
	central	2747.677	25.80	1721.334	40.00	12050.759	38.20	18380.346	39.70	5915.358	35.80
	western	1528.199	28.90	4019.876	13.70	12624.343	13.70	11990.796	16.40	8603.872	13.60
Income level	low	1495.403	29.90	1914.638	30.10	11410.61	27.60	11191.397	30.20	5143.596	30.80
	medium	1680.207	36.90	2482.387	39.90	9630.919	41.00	10985.805	35.40	7656.176	37.20
	high	6177.406	33.10	8758.858	30.00	21359.86	31.40	26879.913	34.30	13215.437	32.00
BOAI	no	3048.229	94.50	3779.979	93.10	14037.244	82.40	18051.785	70.90	8591.743	70.30
	yes	4288.195	5.50	9868.705	6.90	12762.423	17.60	12726.485	29.10	8584.787	29.70
BMI	no	2971.473	90.10	3640.816	84.50	18084.697	33.70	19389.341	35.10	11432.579	38.30
	yes	4435.244	9.90	7226.133	15.50	11618.125	66.30	14940.657	64.90	7004.465	61.70
Living arrangements	self	3534.415	1.70	6086.95	2.10	20976.111	1.80	16895.085	1.60	12845.353	1.70
	family and friends	2867.562	94.00	3948.022	95.00	13088.972	95.50	15887.967	96.50	8123.845	94.00
	government and institutions	8389.505	4.30	11079.271	2.90	33962.433	2.70	47607.088	1.90	18729.447	4.30

Table S4. Multiple linear regression on elderly HCD

Variable	2005 B	2005 SE	2005 t	2005 LLCI	2005 ULCI	2014 B	2014 SE	2014 t	2014 LLCI	2014 ULCI
(Constant)	0.110	0.202	0.544	-0.286	0.505	-1.773***	0.496	-3.575	-2.746	-0.801
gender (female = reference)	-0.386***	0.041	-9.501	-0.466	-0.306	-0.342***	0.087	-3.913	-0.514	-0.171
age	0.068***	0.002	41.231	0.065	0.072	0.092***	0.004	20.991	0.083	0.101
Residence (country = reference)	0.037	0.054	0.696	-0.068	0.142	0.075	0.134	0.561	-0.187	0.337
live type (live alone = reference)										
live with family	0.675***	0.055	12.214	0.566	0.783	0.780***	0.112	6.947	0.560	1.000
live in institution	-0.149	0.155	-0.967	-0.452	0.154	0.351	0.397	0.886	-0.426	1.129
region (western = reference)										
central	0.383***	0.050	7.689	0.285	0.480	0.395***	0.118	3.335	0.163	0.627
eastern	0.518***	0.045	11.506	0.430	0.606	0.404***	0.118	3.435	0.173	0.635
education	0.016***	0.006	2.687	0.004	0.028	0.006	0.013	0.426	-0.020	0.032
household income	0.000***	0.000	-4.116	0.000	0.000	0.000**	0.000	-2.154	0.000	0.000
BOAI (no = reference)	0.058	0.076	0.765	-0.090	0.206	0.021	0.089	0.240	-0.153	0.196
BMI (no = reference)	0.050	0.063	0.793	-0.073	0.173	-0.382***	0.095	-4.038	-0.568	-0.197
living arrangements (self = reference)										
family and friends	0.213	0.142	1.496	-0.066	0.492	0.443	0.318	1.393	-0.181	1.066
government and institutions	1.730***	0.180	9.615	1.377	2.083	1.969***	0.474	4.155	1.040	2.898
children number	0.015*	0.008	1.906	0.000	0.031	0.039*	0.021	1.913	-0.001	0.080
R^2			0.149					0.157		
Prob>F			0.000					0.000		

Note：The level of significance *** P<0.01，** P<0.05，* P<0.1; Abbreviations: B: beta, regression coefficient; SE: standard error; LLCI: lower limit confidence interval; ULCI: upper limit confidence interval. Household income's beta in 2005=$-3.085*10^{-6}$, in 2014=$-2.847*10^{-6}$.

Table S5. Multiple linear regression on elderly HCE

	2005					2014				
**Variable**	**B**	**SE**	**t**	**LLCI**	**ULCI**	**B**	**SE**	**t**	**LLCI**	**ULCI**
(Constant)	2.245***	0.069	32.340	2.109	2.381	3.559***	0.196	18.120	3.173	3.944
gender (female = reference)	-0.031**	0.014	-2.208	-0.058	-0.003	0.018	0.035	0.517	-0.050	0.086
age	-0.001**	0.001	-2.060	-0.002	0.000	-0.005***	0.002	-3.038	-0.009	-0.002
Residence (country = reference)	0.134***	0.018	7.316	0.098	0.170	-0.005	0.053	-0.105	-0.109	0.098
live type (live alone = reference)										
live with family	0.104***	0.019	5.393	0.066	0.142	-0.032	0.045	-0.712	-0.120	0.056
live in institution	-0.084	0.053	-1.587	-0.187	0.020	0.261	0.168	1.549	-0.069	0.591
Region (western = reference)										
central	0.262***	0.017	15.518	0.229	0.295	0.116**	0.046	2.499	0.025	0.207
eastern	0.333***	0.015	21.931	0.303	0.363	0.096**	0.046	2.101	0.006	0.186
education	0.020***	0.002	9.512	0.016	0.024	-0.001	0.005	-0.148	-0.011	0.010
household income	0.000***	0.000	20.026	0.000	0.000	0.000***	0.000	11.286	0.000	0.000
BOAI (no = reference)	0.072***	0.026	2.762	0.021	0.124	-0.108***	0.035	-3.065	-0.178	-0.039
BMI (no = reference)	0.176***	0.021	8.232	0.134	0.218	-0.212***	0.038	-5.613	-0.286	-0.138
living arrangements (self = reference)										
family and friends	0.100**	0.050	1.986	0.001	0.199	0.122	0.126	0.970	-0.125	0.369
government and institutions	0.396***	0.062	6.335	0.273	0.518	0.510***	0.190	2.683	0.137	0.883
children number	0.007***	0.003	2.639	0.002	0.012	0.014*	0.008	1.703	-0.002	0.030
R^2			0.125					0.086		
Prob>F			0.000					0.000		

Note：The level of significance *** P<0.01， ** P<0.05， * P<0.1; Abbreviations: B: beta, regression coefficient; SE: standard error; LLCI: lower limit confidence interval; ULCI: upper limit confidence interval. Household income's beta in 2005=$5.384*10^{-6}$, in 2014=$5.959*10^{-6}$.
